# Supplementary material for: Variant Landscape of 15 Genes Involved in Corneal Dystrophies: Report of 30 Families and Comprehensive Analysis of the Literature
Source: Int J Mol Sci. 2023 Mar 6;24(5):5012. doi: 10.3390/ijms24055012 (PMC10003302; doi:10.3390/ijms24055012)
Supplement: Supplementary file 1 [file ijms-24-05012-s001.zip › ijms-2152855-Supplementary Figures S1- S3.pdf]

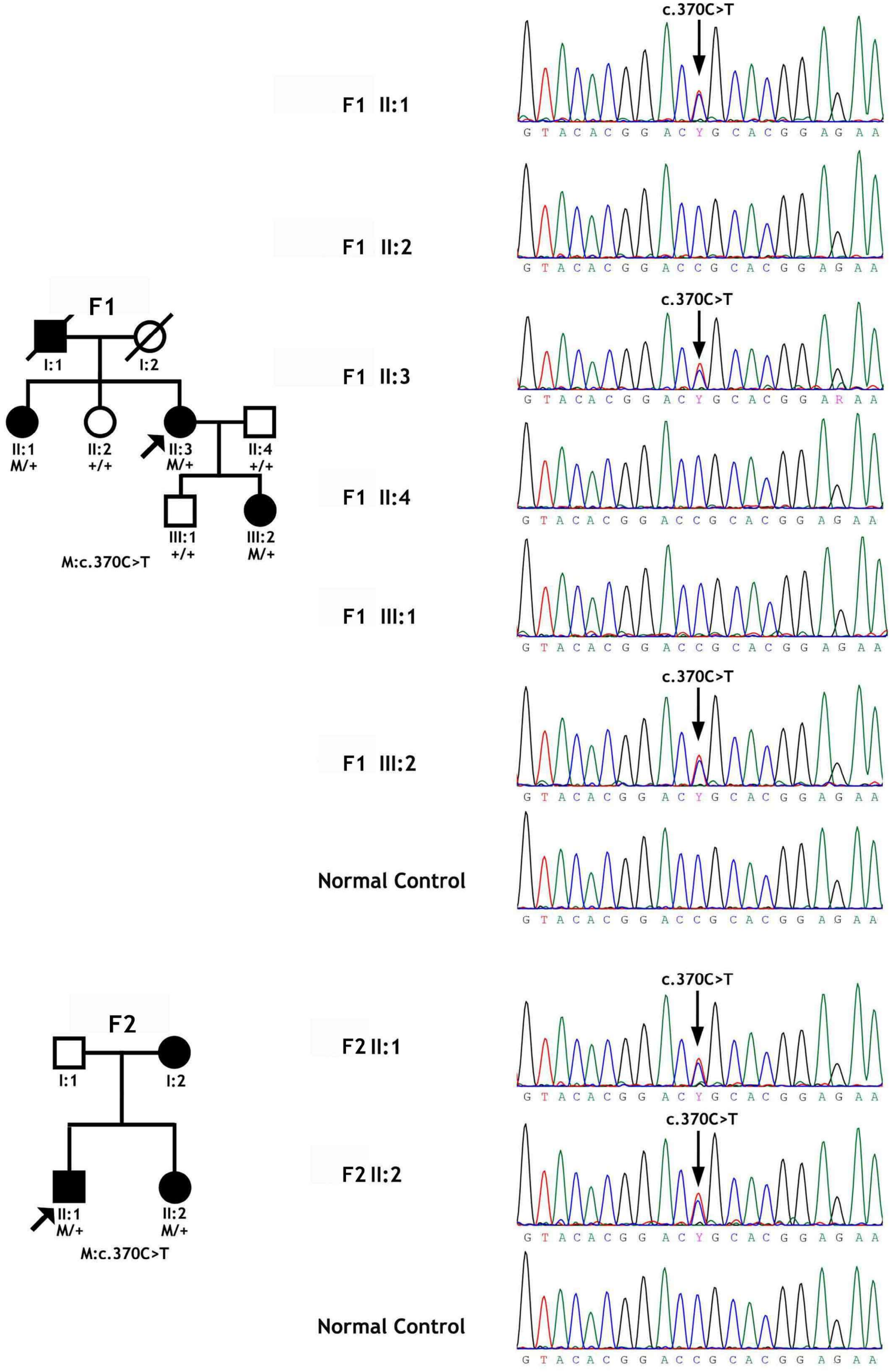

F1
I:1
I:2
II:1
M/+
II:2
+/+
II:3
M/+
II:4
+/+
III:1
+/+
III:2
M/+
M:c.370C>T
F1 II:1
c.370C>T
G T A C A C G G A C Y G C A C G G A G A A
F1 II:2
G T A C A C G G A C C G C A C G G A G A A
F1 II:3
c.370C>T
G T A C A C G G A C Y G C A C G G A R A A
F1 II:4
G T A C A C G G A C C G C A C G G A G A A
F1 III:1
G T A C A C G G A C C G C A C G G A G A A
F1 III:2
c.370C>T
G T A C A C G G A C Y G C A C G G A G A A
Normal Control
G T A C A C G G A C C G C A C G G A G A A
F2
I:1
I:2
II:1
M/+
II:2
M/+
M:c.370C>T
F2 II:1
c.370C>T
G T A C A C G G A C Y G C A C G G A G A A
F2 II:2
c.370C>T
G T A C A C G G A C Y G C A C G G A G A A
Normal Control
G T A C A C G G A C C G C A C G G A G A A

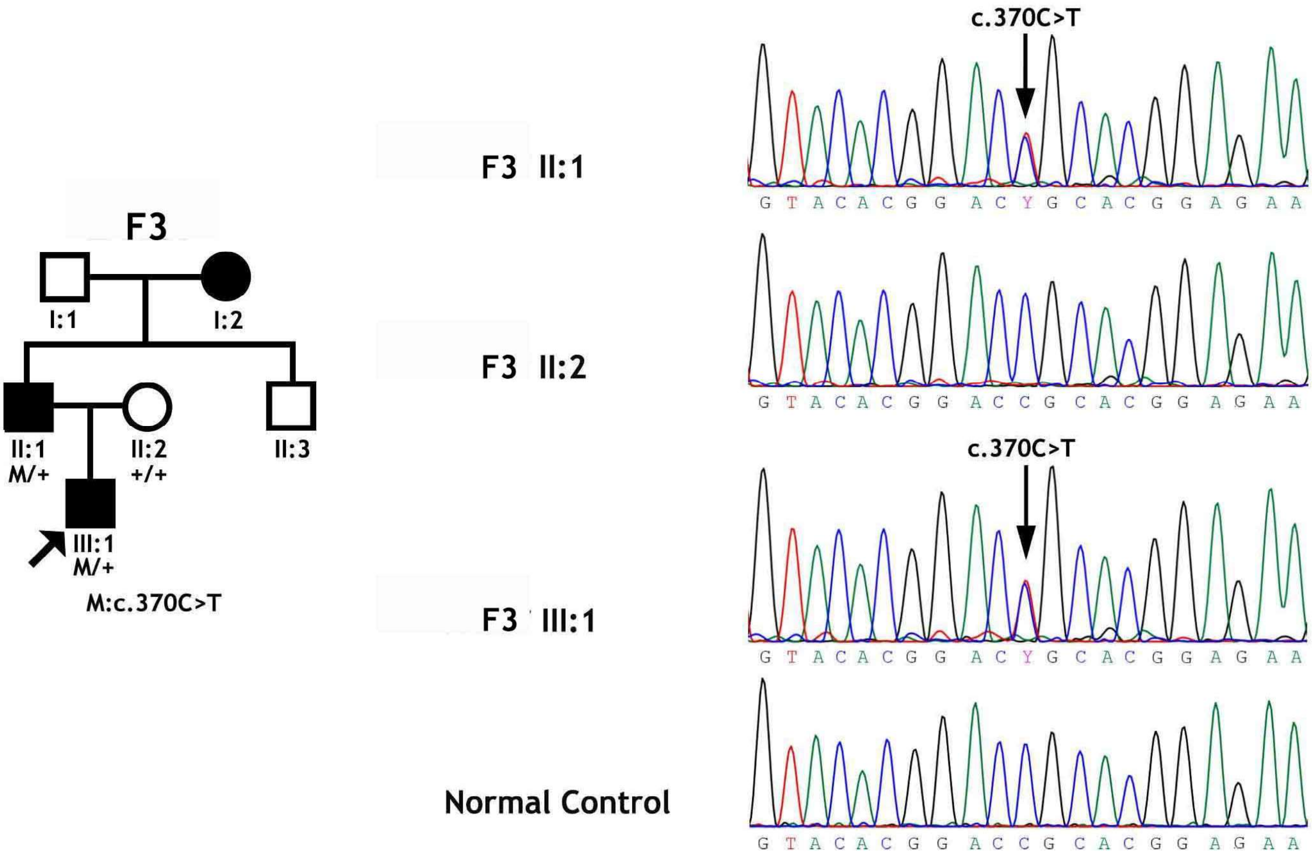
F3
I:1
I:2
II:1
M/+
II:2
+/+
II:3
III:1
M/+
M:c.370C>T
c.370C>T
F3 II:1
G T A C A C G G A C Y G C A C G G A G A A
F3 II:2
G T A C A C G G A C C G C A C G G A G A A
c.370C>T
F3 III:1
G T A C A C G G A C Y G C A C G G A G A A
Normal Control
G T A C A C G G A C C G C A C G G A G A A

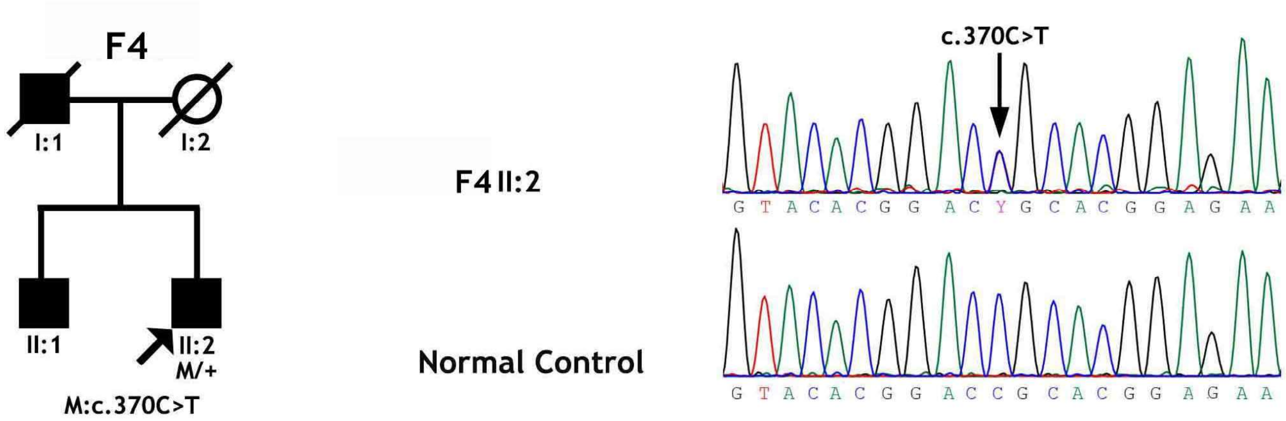
F4
I:1
I:2
II:1
II:2
M/+
M:c.370C>T
c.370C>T
F4 II:2
G T A C A C G G A C Y G C A C G G A G A A
Normal Control
G T A C A C G G A C C G C A C G G A G A A

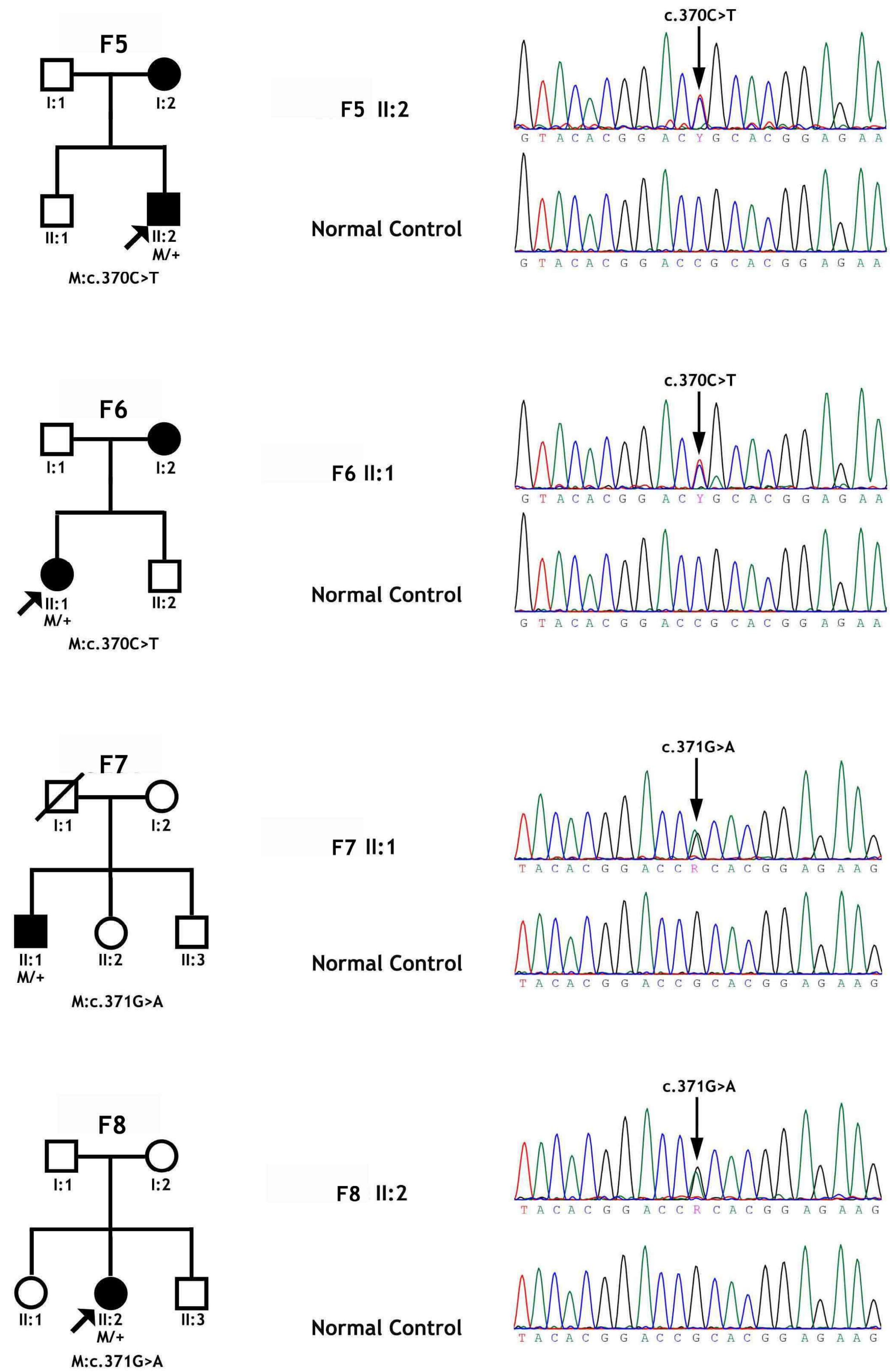

F5
I:1
I:2
II:1
II:2
M/+
M:c.370C>T
c.370C>T
F5 II:2
G T A C A C G G A C Y G C A C G G A G A A
Normal Control
G T A C A C G G A C C G C A C G G A G A A
F6
I:1
I:2
II:1
M/+
II:2
M:c.370C>T
c.370C>T
F6 II:1
G T A C A C G G A C Y G C A C G G A G A A
Normal Control
G T A C A C G G A C C G C A C G G A G A A
F7
I:1
I:2
II:1
M/+
II:2
II:3
M:c.371G>A
c.371G>A
F7 II:1
T A C A C G G A C C R C A C G G A G A A G
Normal Control
T A C A C G G A C C G C A C G G A G A A G
F8
I:1
I:2
II:1
II:2
M/+
II:3
M:c.371G>A
c.371G>A
F8 II:2
T A C A C G G A C C R C A C G G A G A A G
Normal Control
T A C A C G G A C C G C A C G G A G A A G

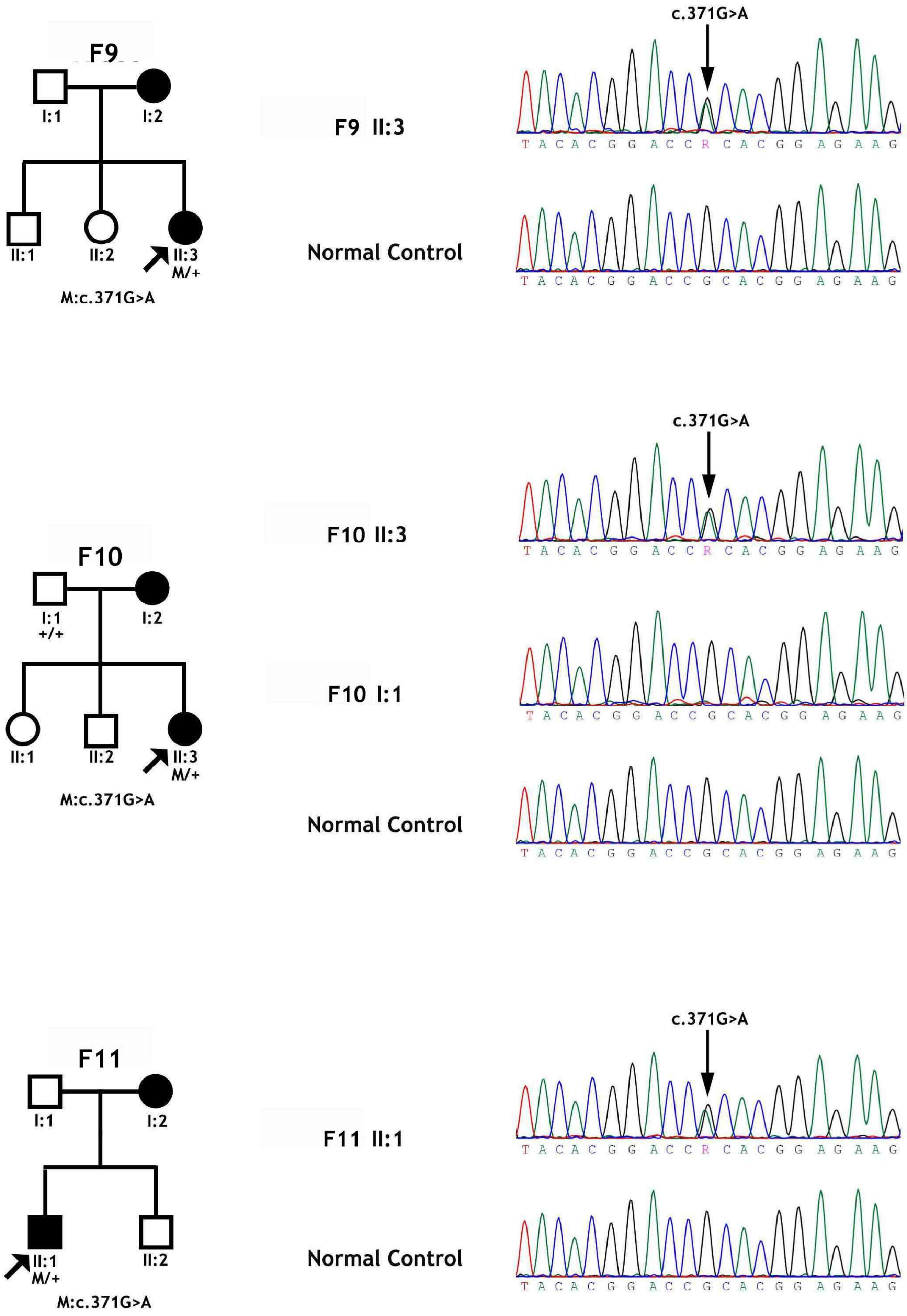
F9
I:1
I:2
II:1
II:2
II:3
M/+
M:c.371G>A
F9 II:3
Normal Control
c.371G>A
T A C A C G G A C C R C A C G G A G A A G
T A C A C G G A C C G C A C G G A G A A G
F10
I:1
+/+
I:2
II:1
II:2
II:3
M/+
M:c.371G>A
F10 II:3
F10 I:1
Normal Control
c.371G>A
T A C A C G G A C C R C A C G G A G A A G
T A C A C G G A C C G C A C G G A G A A G
T A C A C G G A C C G C A C G G A G A A G
F11
I:1
I:2
II:1
M/+
II:2
M:c.371G>A
F11 II:1
Normal Control
c.371G>A
T A C A C G G A C C R C A C G G A G A A G
T A C A C G G A C C G C A C G G A G A A G

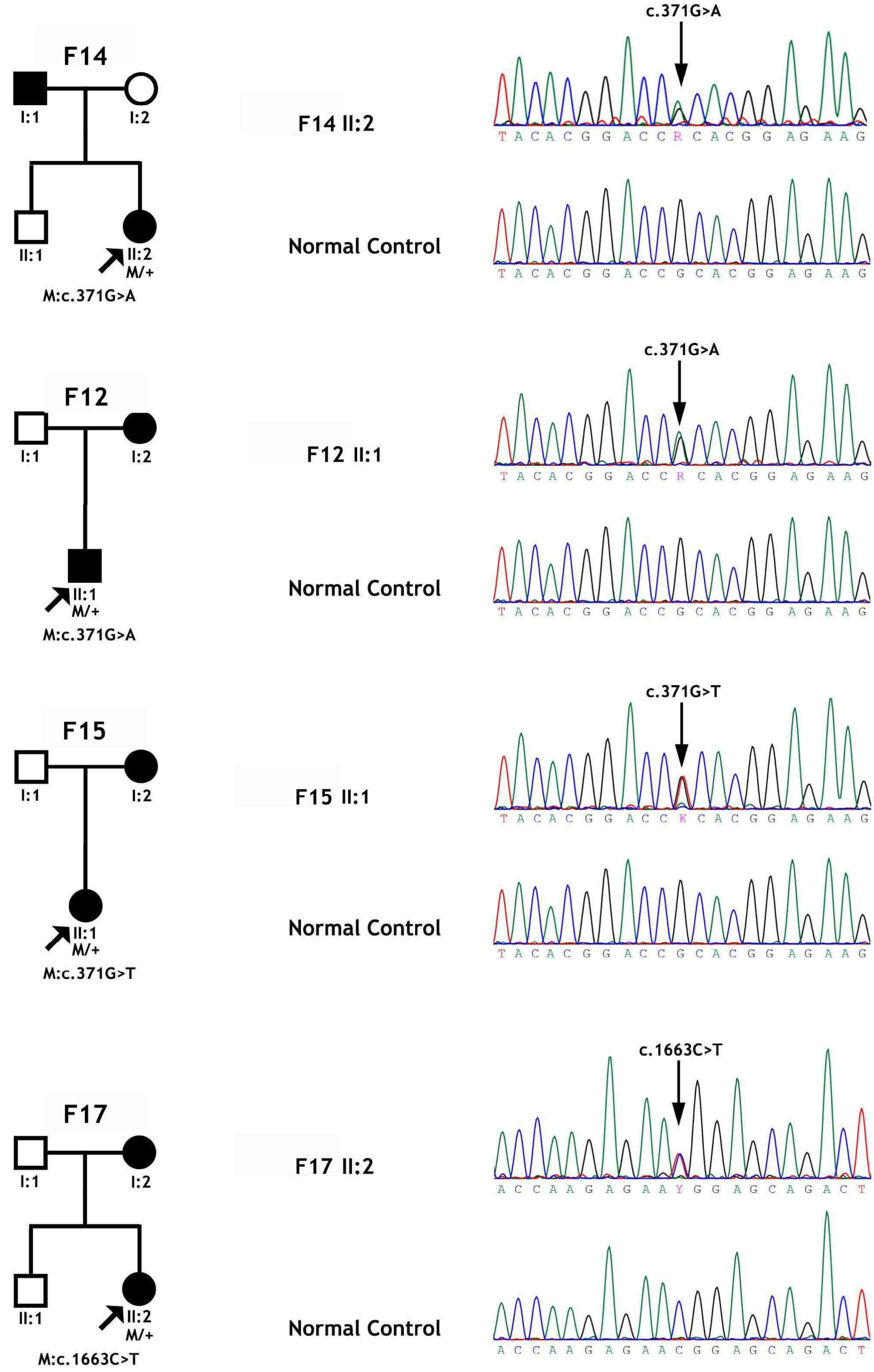

F14
I:1
I:2
II:1
II:2
M/+
M:c.371G>A
F14 II:2
c.371G>A
T A C A C G G A C C R C A C G G A G A A G
Normal Control
T A C A C G G A C C G C A C G G A G A A G
F12
I:1
I:2
II:1
M/+
M:c.371G>A
F12 II:1
c.371G>A
T A C A C G G A C C R C A C G G A G A A G
Normal Control
T A C A C G G A C C G C A C G G A G A A G
F15
I:1
I:2
II:1
M/+
M:c.371G>T
F15 II:1
c.371G>T
T A C A C G G A C C K C A C G G A G A A G
Normal Control
T A C A C G G A C C G C A C G G A G A A G
F17
I:1
I:2
II:1
II:2
M/+
M:c.1663C>T
F17 II:2
c.1663C>T
A C C A A G A G A A Y G G A G C A G A C T
Normal Control
A C C A A G A G A A C G G A G C A G A C T

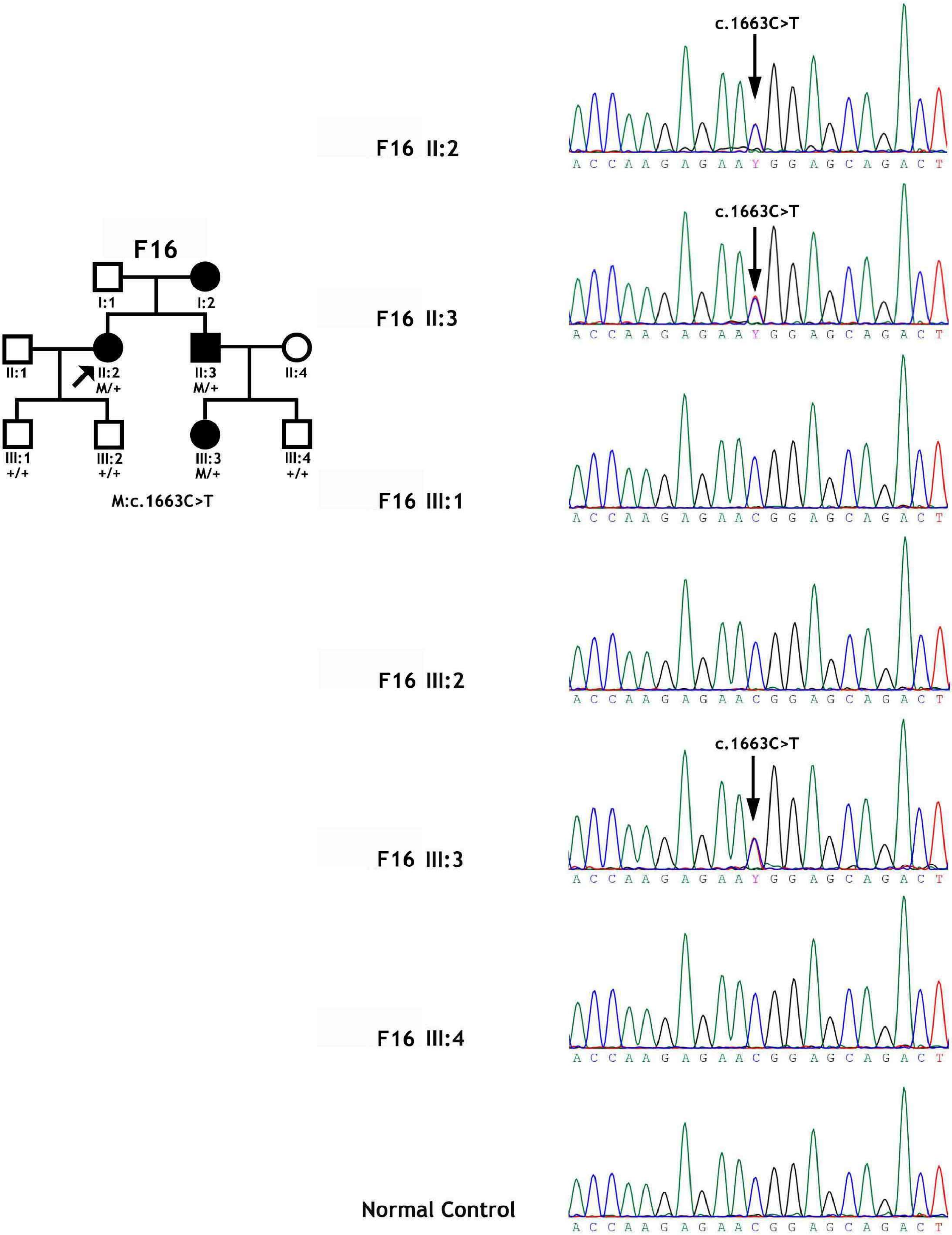

F16
I:1
I:2
II:1
II:2
M/+
II:3
M/+
II:4
III:1
+/+
III:2
+/+
III:3
M/+
III:4
+/+
M:c.1663C>T
c.1663C>T
F16 II:2
A C C A A G A G A A Y G G A G C A G A C T
c.1663C>T
F16 II:3
A C C A A G A G A A Y G G A G C A G A C T
F16 III:1
A C C A A G A G A A C G G A G C A G A C T
F16 III:2
A C C A A G A G A A C G G A G C A G A C T
c.1663C>T
F16 III:3
A C C A A G A G A A Y G G A G C A G A C T
F16 III:4
A C C A A G A G A A C G G A G C A G A C T
Normal Control
A C C A A G A G A A C G G A G C A G A C T

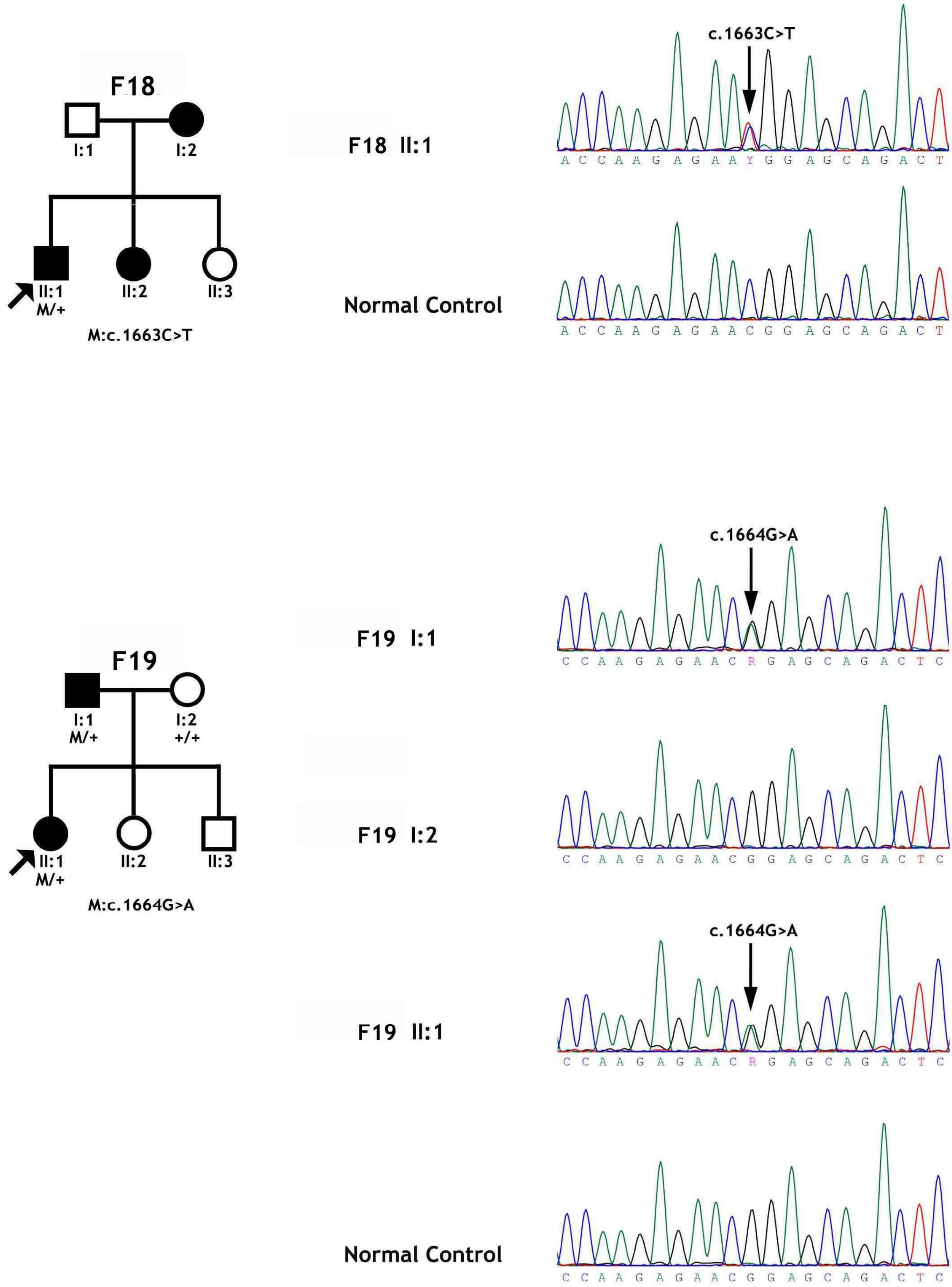
F18
I:1
I:2
II:1
M/+
II:2
II:3
M:c.1663C>T
F18 II:1
c.1663C>T
A C C A A G A G A A Y G G A G C A G A C T
Normal Control
A C C A A G A G A A C G G A G C A G A C T
F19
I:1
M/+
I:2
+/+
II:1
M/+
II:2
II:3
M:c.1664G>A
F19 I:1
c.1664G>A
C C A A G A G A A C R G A G C A G A C T C
F19 I:2
C C A A G A G A A C G G A G C A G A C T C
F19 II:1
c.1664G>A
C C A A G A G A A C R G A G C A G A C T C
Normal Control
C C A A G A G A A C G G A G C A G A C T C

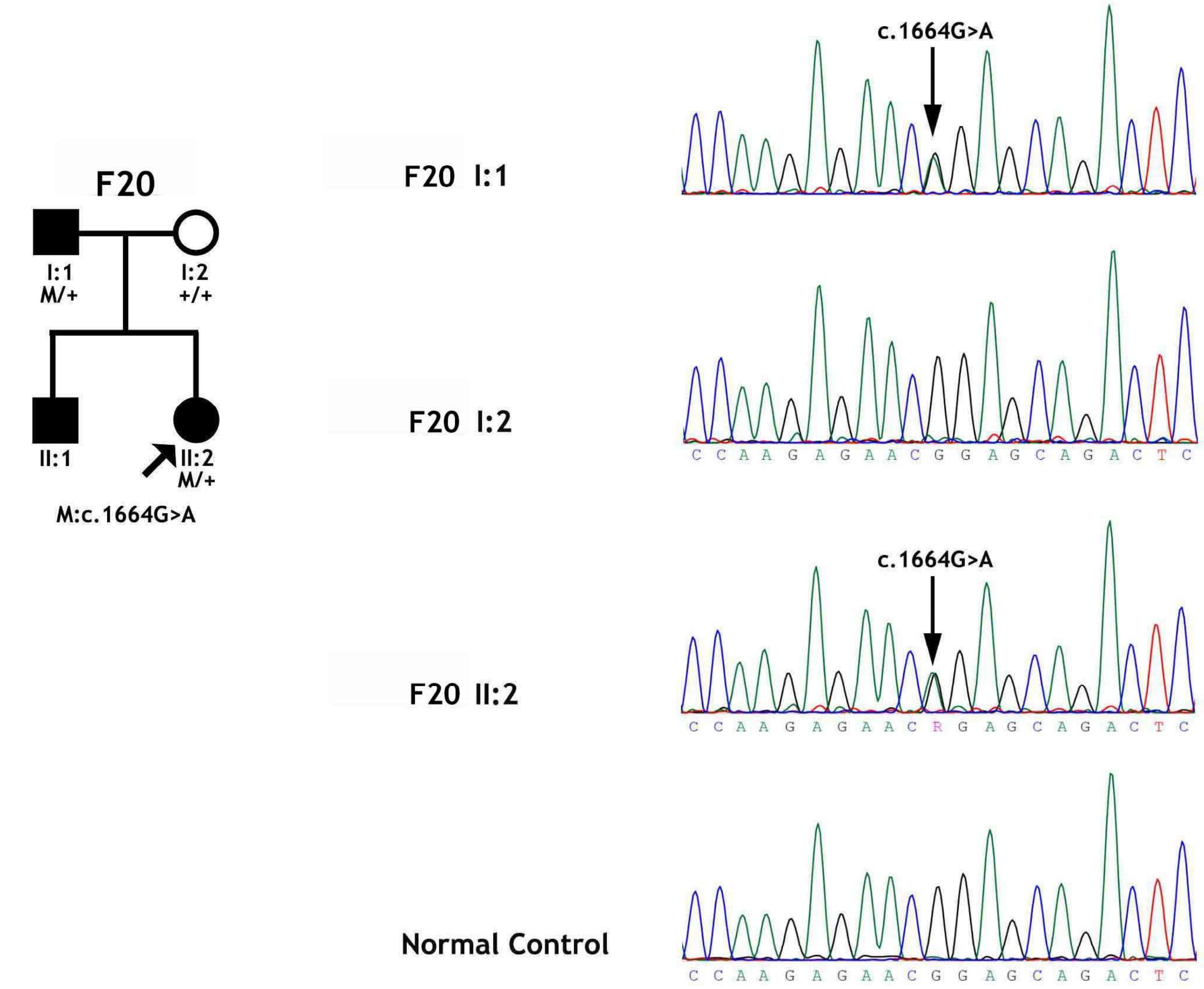
F20
I:1
M/+
I:2
+/+
II:1
II:2
M/+
M:c.1664G>A
F20 I:1
c.1664G>A
F20 I:2
C C A A G A G A A C G G A G C A G A C T C
F20 II:2
c.1664G>A
C C A A G A G A A C R G A G C A G A C T C
Normal Control
C C A A G A G A A C G G A G C A G A C T C

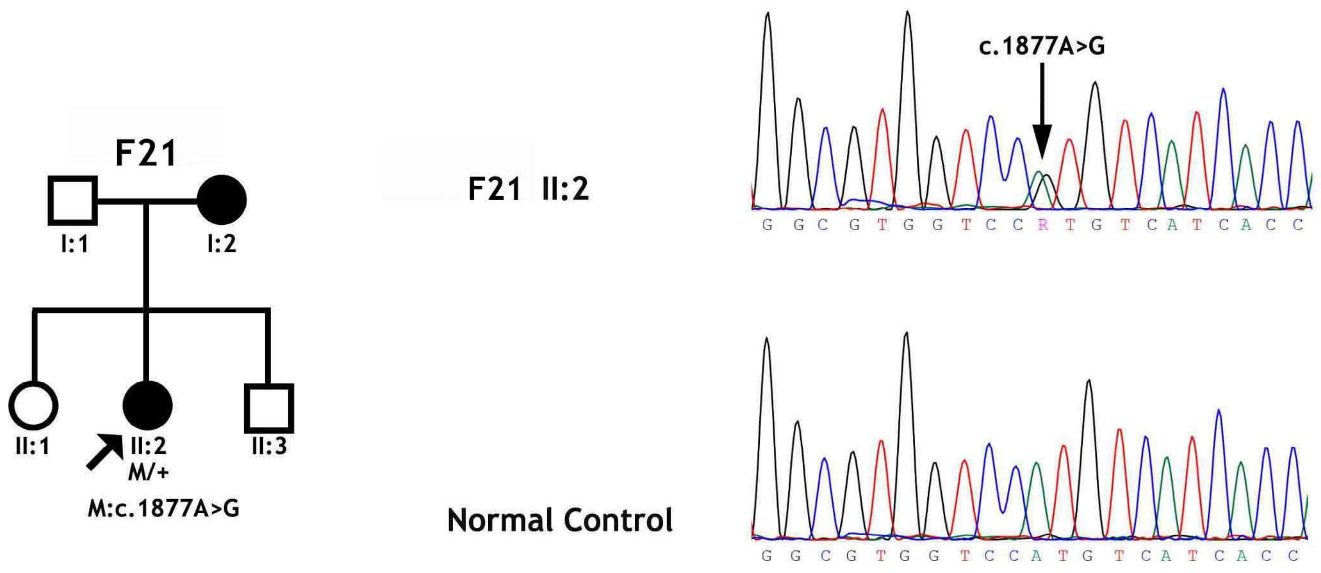
F21
I:1
I:2
II:1
II:2
M/+
II:3
M:c.1877A>G
F21 II:2
c.1877A>G
G G C G T G G T C C R T G T C A T C A C C
Normal Control
G G C G T G G T C C A T G T C A T C A C C

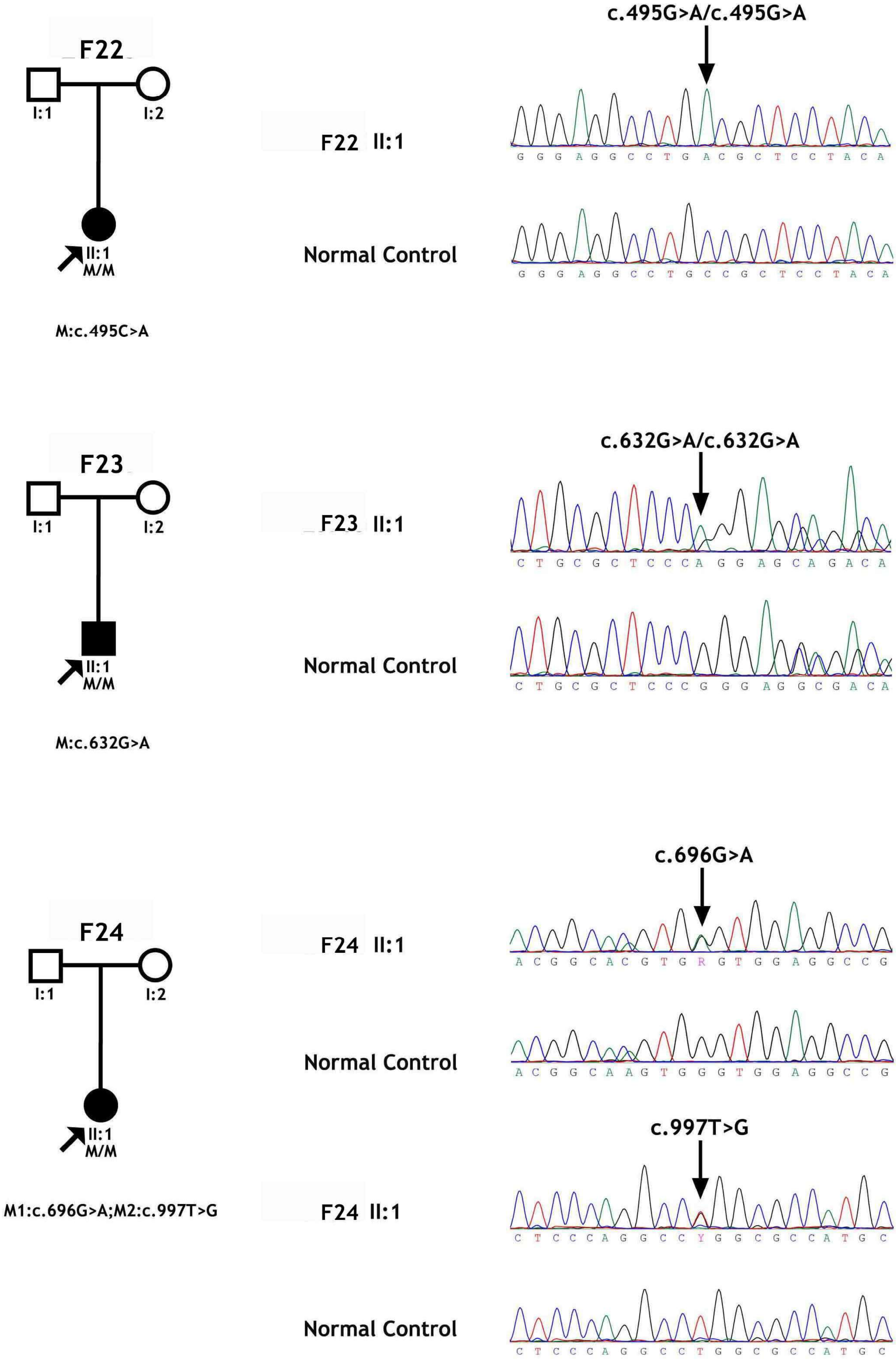

F22
I:1
I:2
II:1
M/M
M:c.495C>A
c.495G>A/c.495G>A
F22 II:1
G G G A G G C C T G A C G C T C C T A C A
Normal Control
G G G A G G C C T G C C G C T C C T A C A
F23
I:1
I:2
II:1
M/M
M:c.632G>A
c.632G>A/c.632G>A
F23 II:1
C T G C G C T C C C A G G A G C A G A C A
Normal Control
C T G C G C T C C C G G G A G G C G A C A
F24
I:1
I:2
II:1
M/M
M1:c.696G>A;M2:c.997T>G
c.696G>A
F24 II:1
A C G G C A C G T G R G T G G A G G C C G
Normal Control
A C G G C A A G T G G G T G G A G G C C G
c.997T>G
F24 II:1
C T C C C A G G C C Y G G C G C C A T G C
Normal Control
C T C C C A G G C C T G G C G C C A T G C

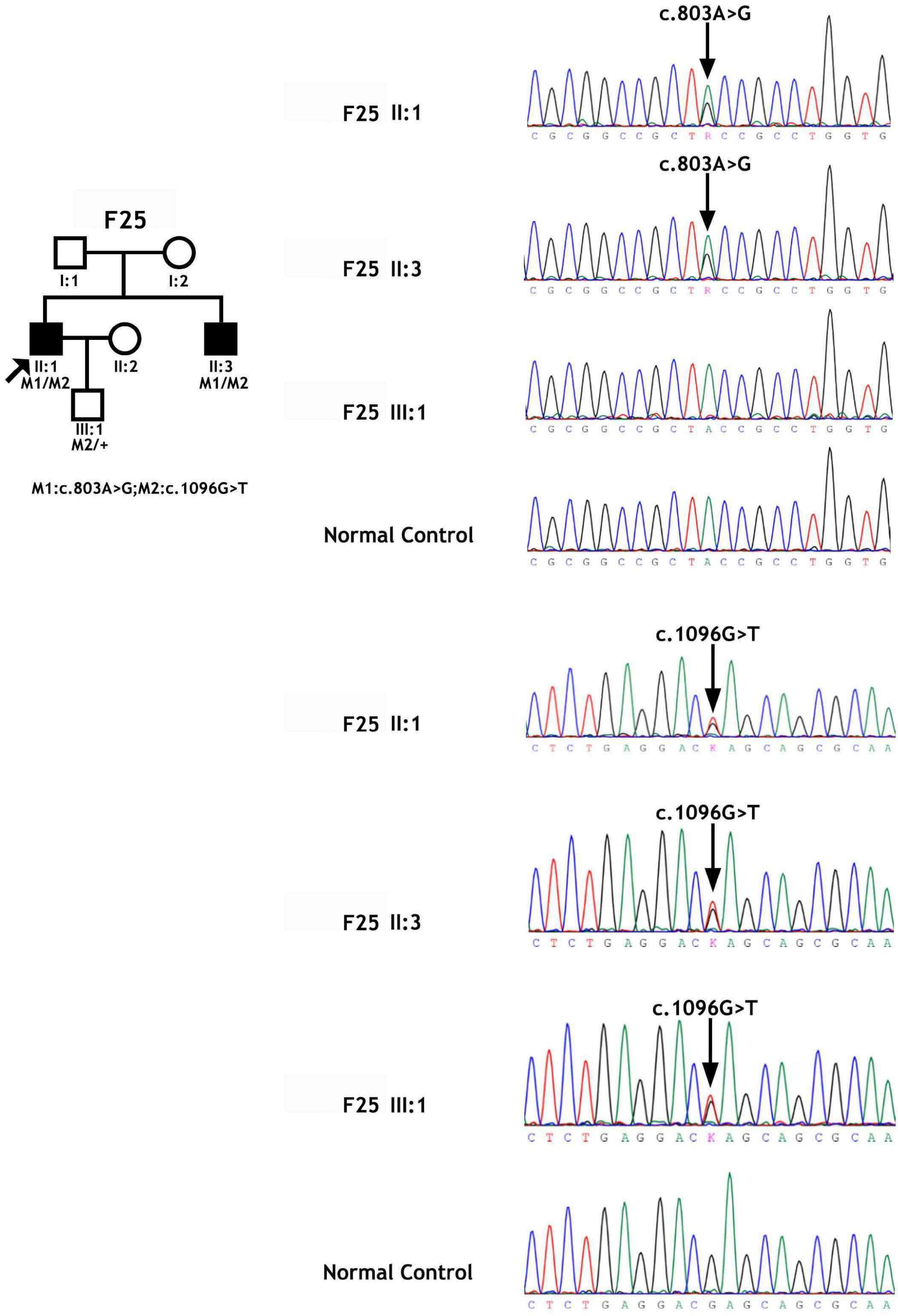

F25
I:1
I:2
II:1
M1/M2
II:2
II:3
M1/M2
III:1
M2/+
M1:c.803A>G;M2:c.1096G>T
c.803A>G
F25 II:1
C G C G G C C G C T R C C G C C T G G T G
c.803A>G
F25 II:3
C G C G G C C G C T R C C G C C T G G T G
F25 III:1
C G C G G C C G C T A C C G C C T G G T G
Normal Control
C G C G G C C G C T A C C G C C T G G T G
c.1096G>T
F25 II:1
C T C T G A G G A C K A G C A G C G C A A
c.1096G>T
F25 II:3
C T C T G A G G A C K A G C A G C G C A A
c.1096G>T
F25 III:1
C T C T G A G G A C K A G C A G C G C A A
Normal Control
C T C T G A G G A C G A G C A G C G C A A

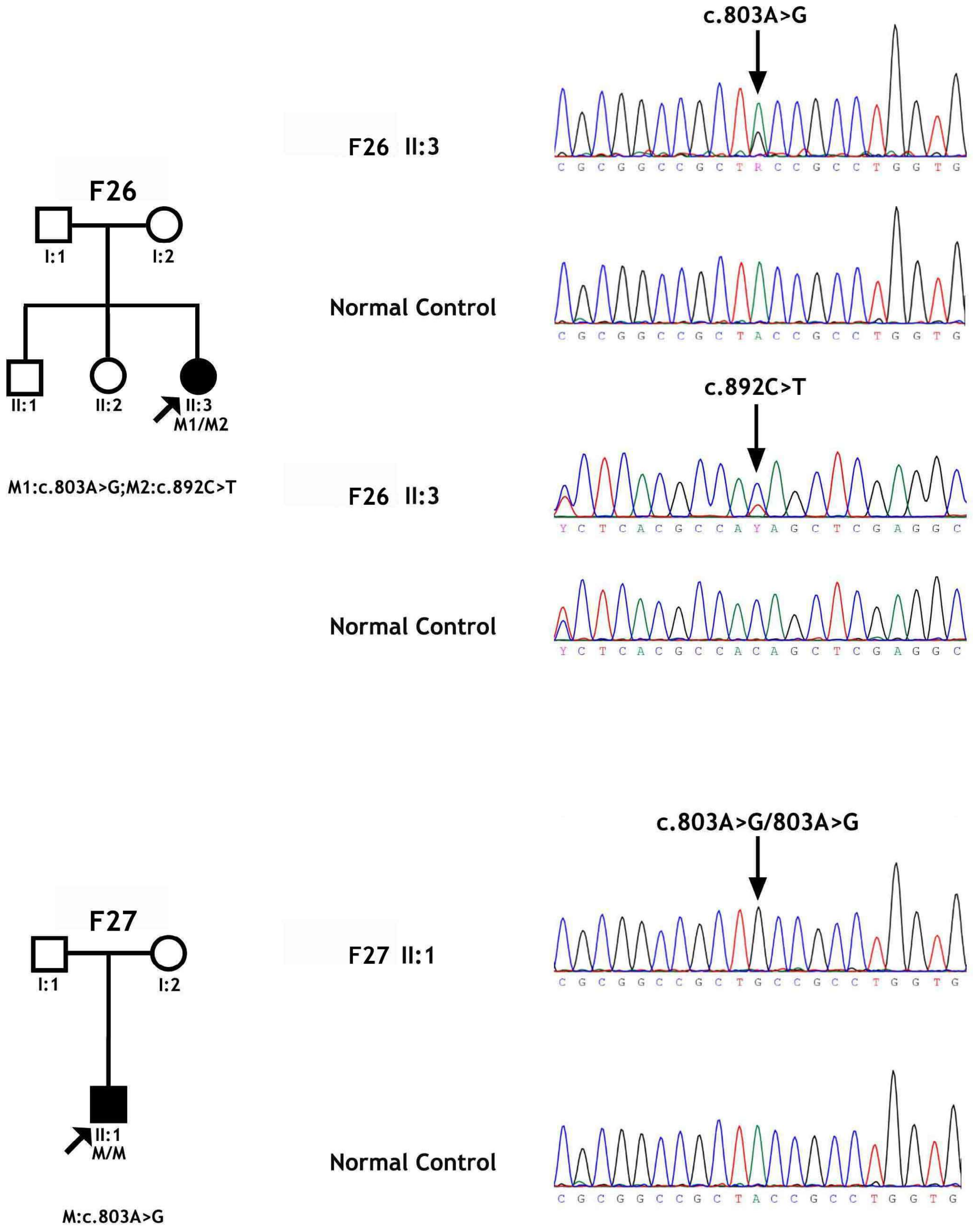
c.803A>G
F26 II:3
C G C G G C C G C T R C C G C C T G G T G
F26
I:1
I:2
Normal Control
C G C G G C C G C T A C C G C C T G G T G
II:1
II:2
II:3
M1/M2
c.892C>T
M1:c.803A>G;M2:c.892C>T
F26 II:3
Y C T C A C G C C A Y A G C T C G A G G C
Normal Control
Y C T C A C G C C A C A G C T C G A G G C
c.803A>G/803A>G
F27
F27 II:1
C G C G G C C G C T G C C G C C T G G T G
I:1
I:2
II:1
M/M
Normal Control
C G C G G C C G C T A C C G C C T G G T G
M:c.803A>G

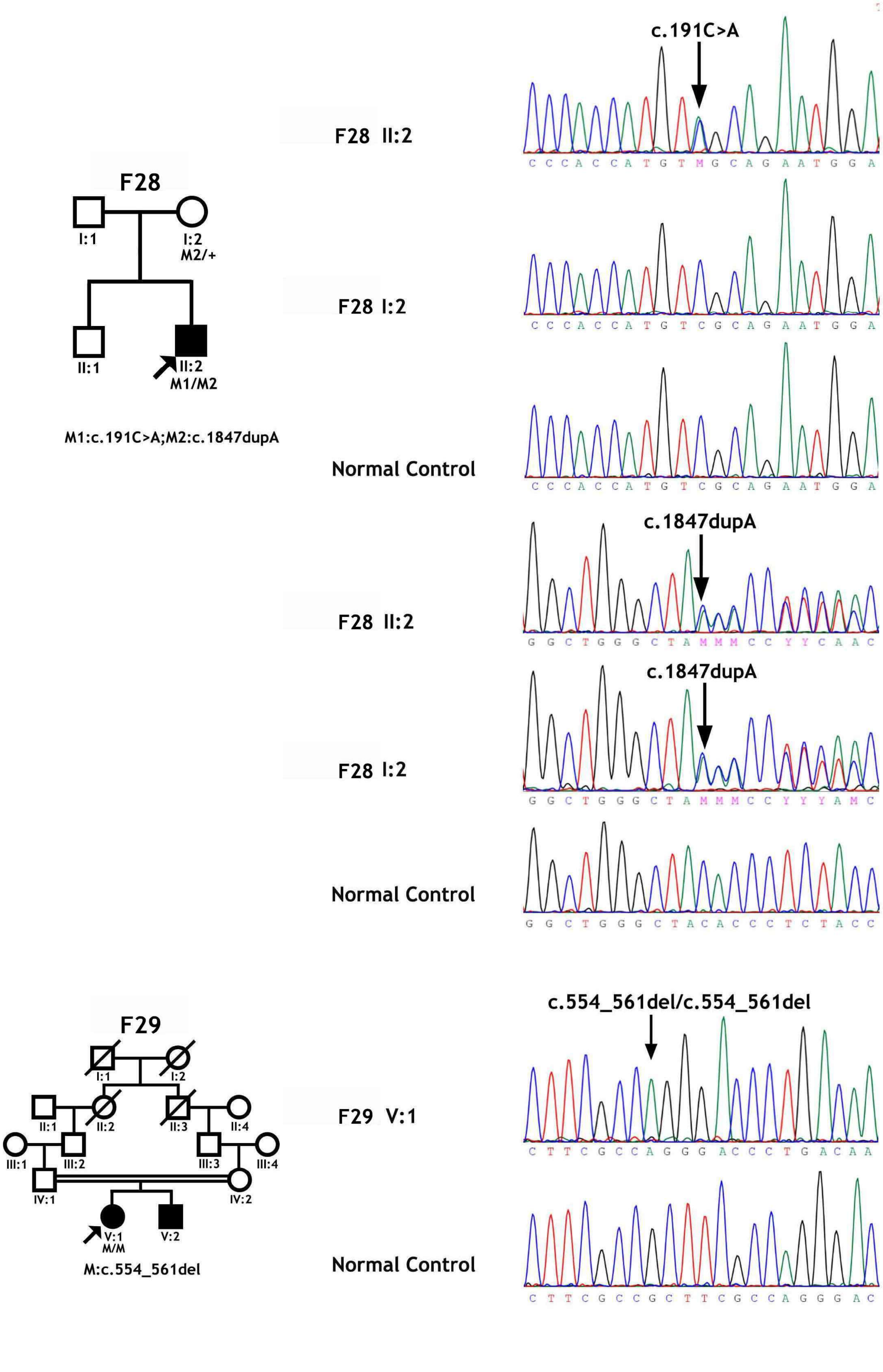
F28
I:1
I:2
M2/+
II:1
II:2
M1/M2
M1:c.191C>A;M2:c.1847dupA
c.191C>A
F28 II:2
C C C A C C A T G T M G C A G A A T G G A
F28 I:2
C C C A C C A T G T C G C A G A A T G G A
Normal Control
C C C A C C A T G T C G C A G A A T G G A
c.1847dupA
F28 II:2
G G C T G G G C T A M M M C C Y Y C A A C
c.1847dupA
F28 I:2
G G C T G G G C T A M M M C C Y Y Y A M C
Normal Control
G G C T G G G C T A C A C C C T C T A C C
F29
I:1
I:2
II:1
II:2
II:3
II:4
III:1
III:2
III:3
III:4
IV:1
IV:2
V:1
M/M
V:2
M:c.554_561del
c.554_561del/c.554_561del
F29 V:1
C T T C G C C A G G G A C C C T G A C A A
Normal Control
C T T C G C C G C T T C G C C A G G G A C

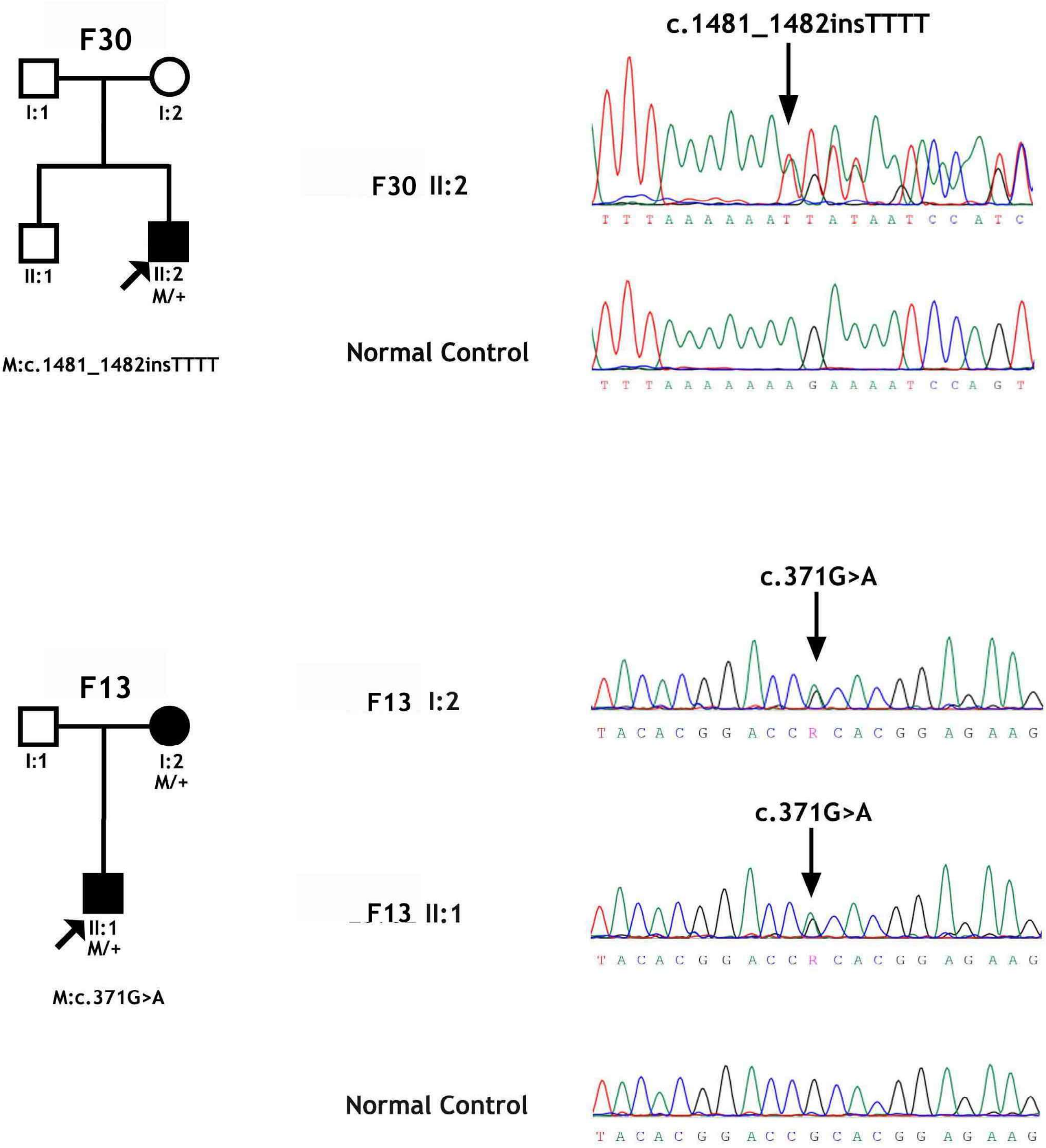

Supplementary Figure S1. Result of Sanger sequencing of in-house families.

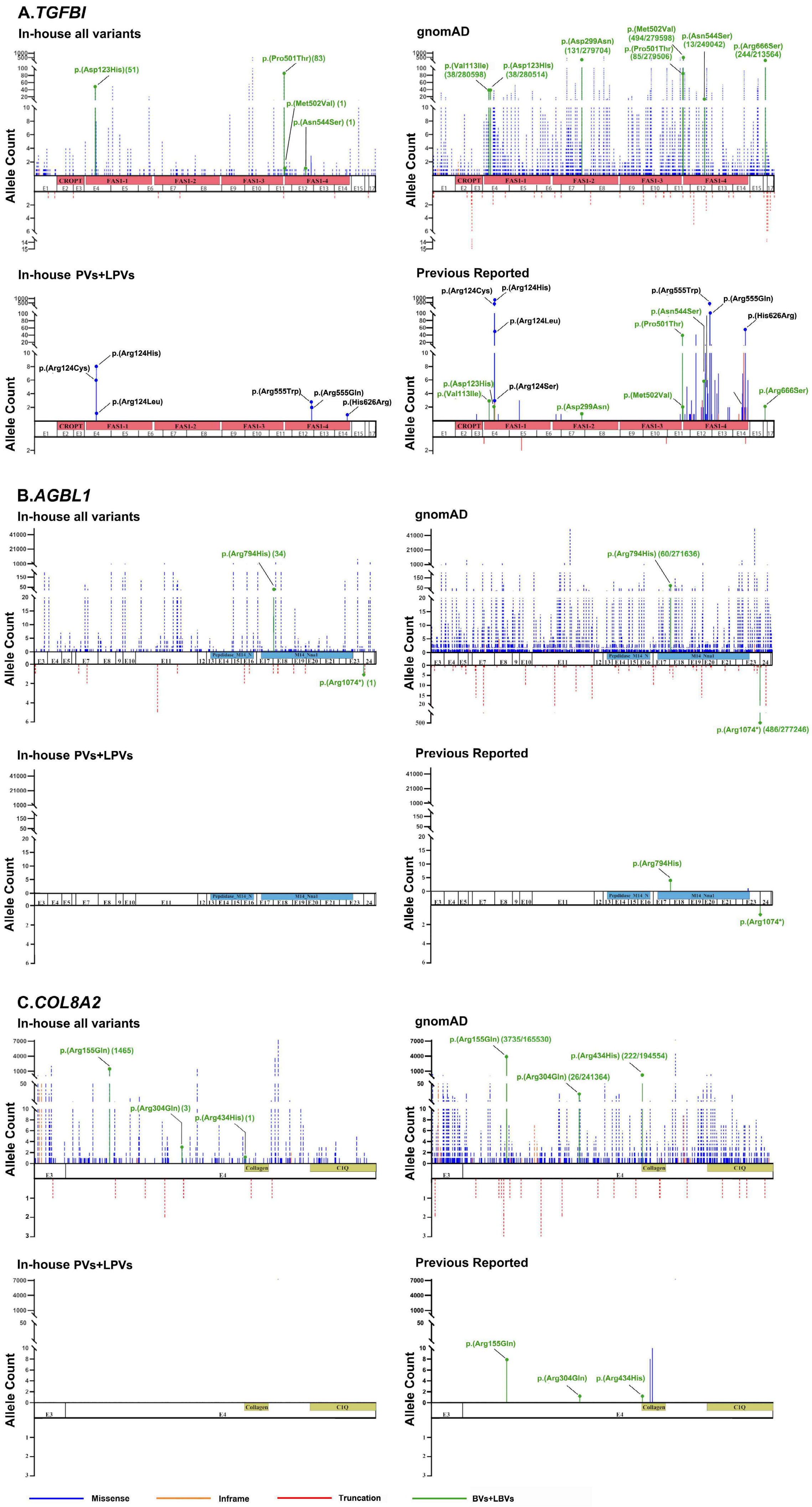
A.TGFBI
In-house all variants
p.(Asp123His)(51)
p.(Pro501Thr)(83)
p.(Met502Val) (1)
p.(Asn544Ser) (1)
Allele Count
CROPT
FAS1-1
FAS1-2
FAS1-3
FAS1-4
gnomAD
p.(Val113Ile) (38/280598)
p.(Asp123His) (38/280514)
p.(Asp299Asn) (131/279704)
p.(Met502Val) (494/279598)
p.(Pro501Thr) (85/279506)
p.(Asn544Ser) (13/249042)
p.(Arg666Ser) (244/213564)
In-house PVs+LPVs
p.(Arg124His)
p.(Arg124Cys)
p.(Arg124Leu)
p.(Arg555Trp)
p.(Arg555Gln)
p.(His626Arg)
Previous Reported
p.(Arg124Cys)
p.(Arg124His)
p.(Arg124Leu)
p.(Asp123His)
p.(Val113Ile)
p.(Arg124Ser)
p.(Asp299Asn)
p.(Arg555Trp)
p.(Arg555Gln)
p.(Asn544Ser)
p.(Pro501Thr)
p.(His626Arg)
p.(Met502Val)
p.(Arg666Ser)
B.AGBL1
In-house all variants
p.(Arg794His) (34)
p.(Arg1074*) (1)
Pepdidase_M14_N
M14_Nna1
gnomAD
p.(Arg794His) (60/271636)
p.(Arg1074*) (486/277246)
In-house PVs+LPVs
Previous Reported
p.(Arg794His)
p.(Arg1074*)
C.COL8A2
In-house all variants
p.(Arg155Gln) (1465)
p.(Arg304Gln) (3)
p.(Arg434His) (1)
Collagen
C1Q
gnomAD
p.(Arg155Gln) (3735/165530)
p.(Arg434His) (222/194554)
p.(Arg304Gln) (26/241364)
In-house PVs+LPVs
Previous Reported
p.(Arg155Gln)
p.(Arg304Gln)
p.(Arg434His)
Missense
Inframe
Truncation
BVs+LBVs

Supplementary Figure S2. The distribution and frequency of variants in *TGFBI*, *AGBL1* and *COL8A2* in our in-house database and gnomAD database and pathogenic or likely pathogenic variants in our in-house database and previously reported literature.

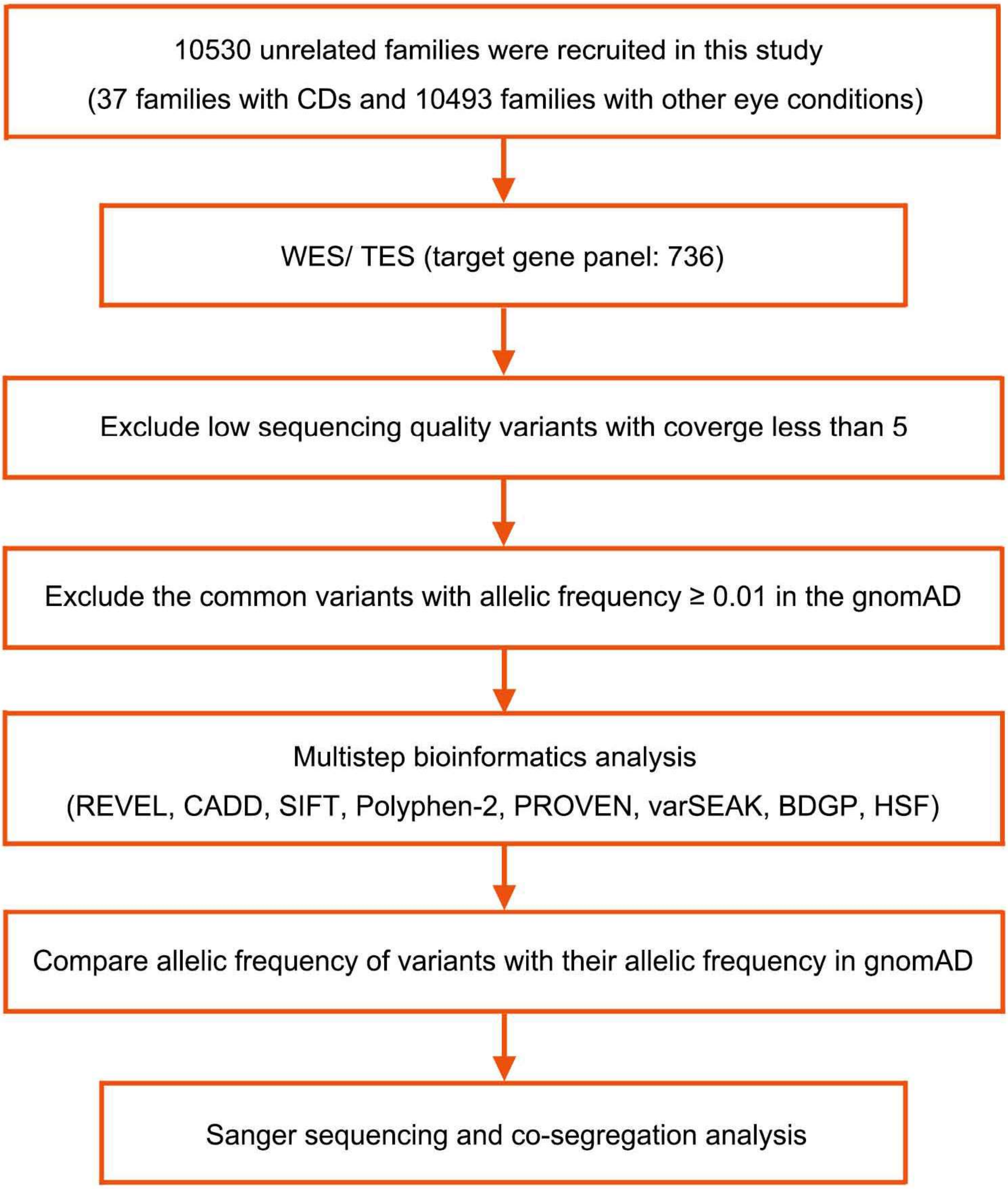

Supplementary Figure S3. Multi-step bioinformatics analysis of variants in our cohort.
